# Advanced Phase Change Materials from Natural Perspectives: Structural Design and Functional Applications

**DOI:** 10.1002/advs.202207652

**Published:** 2023-05-25

**Authors:** Lu Liu, Yuang Zhang, Shufen Zhang, Bingtao Tang

**Affiliations:** ^1^ State Key Laboratory of Fine Chemicals Frontier Science Center for Smart Materials Dalian University of Technology Dalian 116024 P. R. China

**Keywords:** functional applications, natural strategies, phase change materials, structural designs

## Abstract

Phase change materials have garnered extensive interest in heat harvesting and utilization owing to their high energy storage density and isothermal phase transition. Nevertheless, inherent leakage problems and low heat storage efficiencies hinder their widespread utilization. Nature has served as a great source of inspiration for addressing these challenges. Natural strategies are proposed to achieve advanced thermal energy management systems, and breakthroughs are made in recent years. This review focuses on recent advances in the structural design and functions of phase change materials from a natural perspective. By highlighting the structure–function relationship, advanced applications including human motion, medicine, and intelligent thermal management devices are discussed in detail. Finally, the views on the remaining challenges and future prospects are also provided, that is, phase change materials are advancing around the biomimicry design spiral.

## Graphical abstract

The development of nature‐like phase change materials (NPCMs) is a step‐by‐step process for turning natural strategies into sustainable design solutions. Here, recent advancements of NPCMs are summarized for structural design and function integration. On the basis of highlighting the structure‐function relationship, advanced applications are further presented in detail. Exactly, the NPCMs are advancing around the biomimicry design spiral.

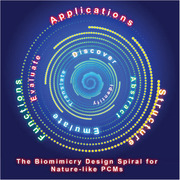

## 1 Introduction

Nature has been, and continues to be, an inexhaustible source of ideas, designs, behaviors, and theories that scientists have always sought to emulate throughout the ages.^[^ ^1^ ^]^ Living organisms in nature embody the perfect unity of structure and function, refined over several hundred million years of evolution.^[^ ^2^ ^]^ Meanwhile, organisms realize optimal energy storage and utilization through close collaboration between structure and function. For example, electric eels,^[^ ^3^ ^]^ the photosynthesis of green plants, and bacteriorhodopsin's efficient photothermal conversion effect,^[^ ^4^ ^]^ have served as great sources of inspiration for mankind in the development and utilization of energy. Typically, thermal energy accounts for more than 80% of the global energy budget and is the dominant source of energy loss.^[^ ^5^ ^]^ Therefore, it is necessary to develop a highly efficient and sustainable thermal‐energy‐utilization strategy. Inspired by natural biological energy storage systems, thermal energy storage (TES) techniques have significantly improved and drawn much attention from both the scientific and industrial communities.^[^ ^6^ ^]^

Currently, phase change materials (PCMs) are drawing great attention as promising TES platforms as the virtue of large energy storage density and isothermal phase transition process.^[^ ^7^ ^]^ Nevertheless, the drawbacks of PCMs, such as leakage problems, phase separation, and supercooling phenomena, resulting in low thermal storage efficiency and a narrow range of applications.^[^ ^8^ ^]^ Here is where the natural strategies have been proposed that offer a path toward solving these challenges.^[^ ^9^ ^]^ Specifically, creatures exhibit physical and chemical features with unique microstructures,^[^ ^10^ ^]^ such as bamboo joints that can effectively retain internal moisture^[^ ^11^ ^]^ and hexagonal honeycombs that possess excellent mechanical properties.^[^ ^12^ ^]^ Natural strategies can also be designed to mitigate the intrinsic drawbacks of PCMs using a combination of bionic strategies and nanoconfinement technology. In addition to the bionic structure of PCMs, it is vital to realize their functional integration by imitating the macro functions of living organisms. Researchers have developed a series of functional, nature‐like PCMs inspired by polar bears,^[^ ^13^ ^]^ cuttlefish, and other creatures,^[^ ^14^ ^]^ which have demonstrated widespread applications. With the continuous optimization of bionic structures and functions, the resulting nature‐like PCMs systems (NPCMs) are excellent candidates for future thermal energy management and regulation.^[^ ^15^ ^]^

With the aim of learning from nature, remarkable advances have been made in the development of NPCMs in recent years; however, a systematic review of the literature is still lacking. In this review, we focus on the recent advances in phase change composites from a natural perspective and provide new insights into achieving smart thermal regulation systems with excellent functions. First, an overview of the state‐of‐the‐art developments in NPCMs is presented in terms of bionic structural design and function coupling. On this basis, we specifically provide detailed insights into the correlation between bionic structural design and function, that is, “the common natural origin and complement to each other,” aiming at exploring a basic guideline for advanced NPCMs design. Next, we outline emerging applications of NPCMs in human motion, medicine, energy conversion, and intelligent thermal management systems. Finally, future prospects and limitations in the development of NPCMs are then provided.

## 2 Overview of PCMs

Thermal energy utilization has always been an important topic in human society, as it relates to almost all aspects of people's daily lives.^[^ ^16^ ^]^ However, the management of thermal energy is challenging owing to its intermittency and discontinuity, resulting in low utilization efficiency.^[^ ^17^ ^]^ PCMs are promising candidates for thermal management, which realize heat energy absorption and release during an isothermal phase transition process.^[^ ^18^ ^]^ As a result, thermal management techniques based on PCMs have become an efficient way to utilize thermal energy, with the benefits of operational simplicity and high energy storage density.

### 2.1 Classification of PCMs

**Figure 1:**
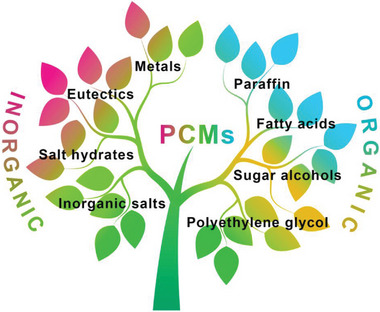
Classification of PCMs based on chemical composition.

PCMs can be divided into inorganic and organic materials according to their chemical composition (**Figure** **1**).^[^ ^19^ ^]^ The abbreviations for the PCMs and additive materials used in this review are summarized in **Table** **1**. Inorganic PCMs were discovered in the 1910s and mainly include water, salts, salt hydrates, and metals, which have the merits of a relatively high energy storage density, good thermal conductivity, and flame retardancy.^[^ ^20^ ^]^ However, there are several hindrances in practical applications, such as supercooling and phase separation, which limit their efficient utilization of thermal energy.^[^ ^21^ ^]^ The development of organic PCMs, including paraffin waxes, fatty acids, and alcohols, started in the 1950s.^[^ ^22^ ^]^ Comparatively, organic PCMs possess a relatively high phase transition enthalpy and are easy to handle. Despite not suffering from phase separation and supercooling, inherent leakage problems, flammability, and low heat storage efficiency have hindered their further development.^[^ ^23^ ^]^ Numerous efforts have been made to overcome these challenges and to significantly progress the development of PCMs.

**Table 1:** Summary of the abbreviations used for PCMs, supporting materials, and additives

Classification		Material	Abbreviation
Phase change materials	Organic materials	Polyethylene glycol	PEG
		Paraffin wax	PW
		Lauric acid	LA
		Stearic acid	SA
	Inorganic materials	Vanadium dioxide	VO_2_
Supporting materials	Organic materials	Polystyrene	PS
		Polyvinylpyrrolidone	PVP
		Polyvinyl alcohol	PVA
		Polyacrylonitrile	PAN
		Cellulose nanocrystals	CNC
		Polymer‐polymethyl methacrylate	PMMA
		Melamine‐formaldehyde	MF
	Inorganic materials	Graphene oxide	GO
		Boron nitride	BN
		Carbon quantum dots	CQDs
		Carbon nanotubes	CNTs
	Organic materials	Imipenem	IMP
Other additives		Doxorubicin hydrochloride	DOX
		Polydopamine	PDA
	Inorganic materials	Zeolitic imidazolate framework‐8	ZIF‐8
		Black phosphorus	BP

### 2.2 Evolution of PCMs

The evolution of PCMs can be traced back to the 20th century and continues with advancements in materials science and technology.^[^ ^24^ ^]^ In this process, the development of PCMs has mainly focused on two aspects: confinement technologies and functionalization. To address the issue of decreased thermal energy storage efficiency owing to leakage problems, confinement technologies have been used to effectively prevent problematic contact between PCMs and their surrounding environments.^[^ ^25^ ^]^ This is achieved by encapsulating PCMs in porous hosts or stabilizing them with nanoparticles or fibers. These strategies can be divided into macro‐, micro‐, and nanoconfinement, according to their different confinement structures.^[^ ^26^ ^]^ The efficient design structure in confinement technology not only mitigates the drawbacks of PCMs but also improves their thermophysical properties.^[^ ^27^ ^]^ Thus, the structural design of PCMs is crucial for confinement. The same is true for the functionalization of PCMs. Thermal conductivity enhancement and energy conversion, as important branches, have become the mainstream trend in PCM research, which involve introducing thermally conductive fillers and energy conversion factors.^[^ ^28^ ^]^ However, additives inevitably reduce the thermal storage ability of PCMs, which can also be compensated for by structural optimization.^[^ ^29^ ^]^ Additionally, PCM functionalization needs to be further explored. Therefore, the rational design of structures and functionalities plays an indispensable role in the development of PCMs. Optimal solutions have always been pursued, and natural strategies have emerged as required. These natural strategies involve obtaining artificial products by mimicking the attributes of creatures, which realize a cooperative effect between structure and function. In view of this, natural strategies are deemed promising approaches to facilitate the development of high‐performance PCMs, which mainly involve two aspects: i) structurally improving PCMs by combining natural structures and nanoconfinement technologies, and ii) integrating nature‐like functionality into PCMs by learning from nature.

## 3 Advances: Learning from Nature

**Figure 2:**
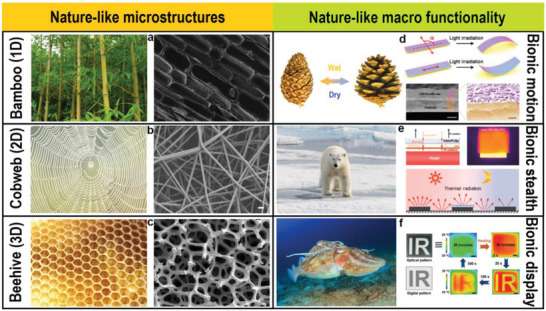
Natural prototypes of NPCMs. a) Perpendicular columnar structure of bamboo joints and NPCMs with a bamboo‐like structure. Reproduced with permission.^[^ ^11b^ ^]^ Copyright 2022, Elsevier. b) Cobweb structure and cobweb‐like structure phase change nanofibers. Reproduced with permission.^[^ ^7c^ ^]^ Copyright 2015, American Chemical Society. c) Beehive microstructures and NPCMs with a beehive‐like structure. Reproduced with permission.^[^ ^9b^ ^]^ Copyright 2022, Elsevier. d) Reversible opening/closing movements of a pinecone and thermal management bionic robots. Reproduced with permission.^[^ ^32^ ^]^ Copyright 2016, American Chemical Society. e) Infrared thermal stealth of polar bear and bionic stealth thermal management systems. Reproduced with permission.^[^ ^34^ ^]^ Copyright 2019, American Chemical Society. f) Coloration dynamic changing behavior of cuttlefish and thermoresponsive NPCM display devices. Reproduced with permission.^[^ ^35^ ^]^ Copyright 2021, Wiley‐VCH.

Nature offers numerous wonderful prototypes for the development of PCMs and has motivated scientists to imitate and transcend them. The ingenious structures of various creatures have been created through natural selection. For example, bamboo joints exhibit tubular structures with inner vertical xylem vessels that are used to efficiently transport nutrients and water (**Figure** **2**a).^[^ ^11^ ^]^ Spiderwebs exhibit high mechanical adaptability owing to their heterogeneous hierarchical structures^[^ ^30^ ^]^ (Figure 2b), and honeycombs with hexagonal close‐packed structures possess excellent mechanical properties (Figure 2c).^[^ ^12^ ^]^ Stimulated by these natural structures, artificial products with mimicking structures have been designed and proven to be effective in overcoming the traditional limitations of materials. Therefore, the use of artificial materials that mimic natural structures has become an attractive and fashionable approach.^[^ ^31^ ^]^ In addition to bionic structural design, nature also inspires new insights into the development of smart composites with wonderful functions. Examples include the opening movements in pinecones (Figure 2d)^[^ ^32^ ^]^ and the self‐protection behaviors of cuttlefish and polar bears (Figure 2e,f).^[^ ^33^ ^]^ Subsequently, a series of systems that mimic natural functions have been developed. The functional bionics of NPCMs have evolved considerably, with technological developments in recent years. There are several key issues that have been exposed in this process despite the great advances that have been made. Under these circumstances, advances in the structure and function of NPCMs are discussed in detail in this section. Finally, the structural design–function relationships are summarized for further optimization.

### 3.1 Nature‐Like Microstructures of Encapsulation Strategy

Biological structures found in nature have achieved nearly flawless performances over billions of years of evolution.^[^ ^36^ ^]^ Such as the delicate ingenious honeycomb structure possesses excellent mechanical properties and high porosity, and the meticulous cobweb structure exhibits strong adhesion and tensile abilities.^[^ ^37^ ^]^ As expected, these natural prototypes introduced new opportunities for the exploration of high‐performance nanocomposites.^[^ ^38^ ^]^ In this respect, nature also brings profound inspiration for the confinement of PCMs. For example, Zhang et al. combined spinning confinement technology with spider silk structure to fabricate PCM fibers with super mechanical strength and elasticity.^[^ ^39^ ^]^ In another work, Yang, and co‐workers incorporated a sponge structure into a porous encapsulation strategy to better coordinate thermal energy storage and heat diffusion abilities.^[^ ^40^ ^]^ Other examples include cells, bamboo, fish, birds, and plants with various structures.^[^ ^41^ ^]^ It is evident that the combination of natural structures and confinement technology can not only mitigate the intrinsic drawbacks of PCMs but also improve their thermodynamic properties.^[^ ^42^ ^]^ Consequently, nature‐inspired structural confinement strategies can be categorized into i) cell‐like structure microcapsule confinement, ii) plants‐like structure porous confinement, and iii) cobweb‐like structure fibrous confinement. In this part, the confinement strategies of NPCMs are summarized in detail.

#### 3.1.1 Cell‐Like Structure Microcapsule Confinement

**Figure 3:**
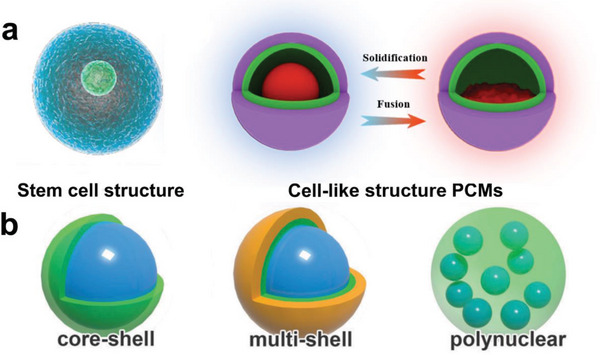
Cell‐like structure microcapsule confinement. a) Schematic illustration of cell‐like structure microcapsule confinement. b) Structural diagrams of various microcapsules with cell‐like structures. Reproduced with permission.^[^ ^6b^ ^]^ Copyright 2018, Royal Society of Chemistry.

The discovery and imitation of biological cell structures have sparked great interest among both scientific and industrial communities.^[^ ^43^ ^]^ In particular, the formation of an intracellular compartment structure realizes structural and functional complexity.^[^ ^44^ ^]^ It also brings tremendous inspiration in the development of shape‐stabilized PCMs (**Figure** **3**a). In the 1950s, Barret K Green first discovered and developed a microencapsulation technique.^[^ ^45^ ^]^ Since then, microencapsulation has been a widely used PCM encapsulation strategy and has played a vital role in its commercialization.^[^ ^46^ ^]^ In general, solid particles or liquid droplet PCMs as the “nucleus” and are enveloped with the “cell membrane” protective shell material.^[^ ^47^ ^]^ To date, numerous shell materials have been reported, including organic polymers, silica, metal oxides, and hybrid materials.^[^ ^48^ ^]^ The incorporation of shell materials not only improves the physical and chemical stability of microcapsules but also endows them with multifunctional properties.^[^ ^49^ ^]^ Based on their structure, cell‐like structure PCMs can be categorized as single core‐shell, multi‐shell, and polynuclear microcapsules (Figure 3b).

**Figure 4:**
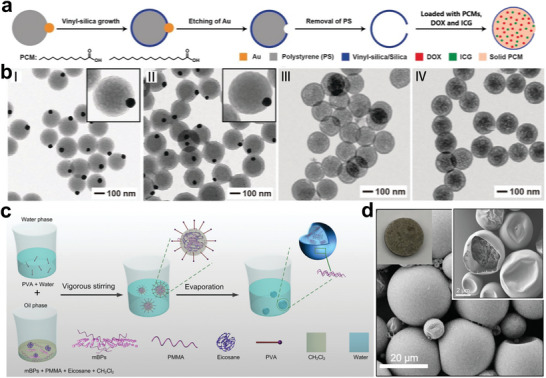
Single core‐shell NPCMs. a) Template synthesis schematic of core‐shell PCMs. b) TEM images of (I–III) silica nanocapsules and (IV) core‐shell NPCMs. Reproduced with permission.^[^ ^51^ ^]^ Copyright 2019, Wiley‐VCH. c,d) Schematic illustration of mBPs‐NPCMs preparation and corresponding SEM images. Reproduced with permission.^[^ ^55^ ^]^ Copyright 2020, Wiley‐VCH.

Emulsion, interfacial, and in situ polymerization are the most common techniques for synthesizing cell‐like structure PCMs.^[^ ^50^ ^]^ Recently, Xia et al. prepared core‐shell PCMs with temperature‐controlled drug release.^[^ ^51^ ^]^ As shown in **Figure** **4**a, silica‐based nanocapsules were fabricated by templating Au‐polystyrene (PS) Janus colloidal particles. After removing the template, a well‐defined hole was observed in the walls of the silica‐based microcapsules (Figure 4b). Subsequently, fatty acids, the therapeutic drug, and near‐infrared (NIR) dye were all loaded into the microcapsules through a hole under vacuum impregnation. When exposed to photoirradiation, payloads can be released through the hole as the fatty acids melt. The core‐shell structure endows PCMs with numerous advantages, including enhanced stability and the ability to load multifunctional components.^[^ ^52^ ^]^ In this case, the performance and applications of cell‐like structure PCMs mainly depend on the characteristics of the shell material.^[^ ^53^ ^]^ Hence, the fabrication of cell‐like structure PCMs with hybridized shells has gained attention in recent years.^[^ ^54^ ^]^ As depicted in Figure 4c,d, Yu, and co‐workers designed an efficient solar energy storage microcapsule PCMs utilizing the emulsion polymerization method, which encapsulates eicosane and modified black phosphorus sheets (mBPs) with high light transmittance shell polymer‐polymethyl methacrylate (PMMA).^[^ ^55^ ^]^ The maximum encapsulation efficiency of eicosane was greater than 78%, and the latent heat exceeded 180 J g^−1^. In addition to their high thermal reliability, the obtained microcapsule PCMs exhibited excellent photothermal conversion properties incorporated with BPs.

**Figure 5:**
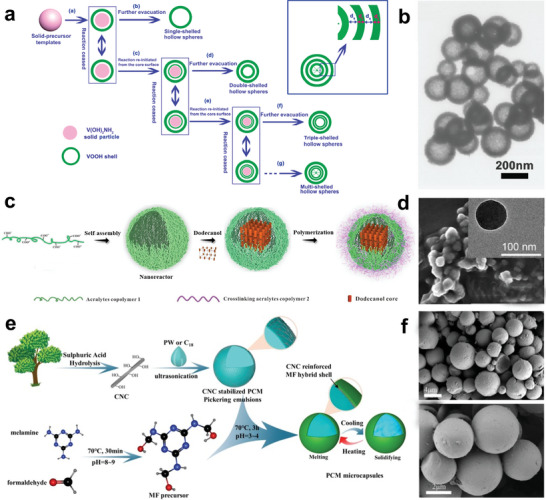
Multi‐shell and polynuclear NPCMs. a) Schematic outline of multi‐shelled microcapsules prepared using solid‐template methods. b) TEM images of double‐shelled microcapsules. Reproduced with permission.^[^ ^58^ ^]^ Copyright 2009, American Chemical Society. c,d) Synthesis route of the NPCMs and SEM images. Reproduced with permission.^[^ ^59^ ^]^ Copyright 2020, Elsevier. e,f) Schematic illustration of CNC/MF NPCMs and corresponding SEM images. Reproduced with permission.^[^ ^60^ ^]^ Copyright 2022, Elsevier.

In comparison with the single core‐shell structure, multi‐shell microcapsules possess the merits of greater structural stability and improved mechanical properties.^[^ ^6b^ ^]^ On the other hand, the complexity of the structure implies greater difficulty in the preparation techniques.^[^ ^56^ ^]^ To this end, numerous efforts have been made to develop stepwise polymerization, layer‐by‐layer templating, and other preparation methods.^[^ ^57^ ^]^ For instance, Xie et al. proposed a method to synthesize hollow structures of multiple shells (**Figure** **5**a,b) by modulating the reaction temperature. They used V(OH)_2_NH_2_ as a solid template and fabricated hollow structures through multiple processes.^[^ ^58^ ^]^ The PCMs (CaCl_2_·6H_2_O) were then infiltrated into the hollow structure via vacuum impregnation. Compared with single‐shell PCMs, these microcapsules exhibited better cyclic energy storage performance. The formation of a multiple‐shell structure is conducive to overcoming the phase separation and supercooling issues. In addition, multi‐shell structures play an important role in improving mechanical properties by modifying the microcapsule interface. Recently, Wang and co‐workers developed a supramolecular lock‐layer technique to obtain nanoencapsulated core‐shell PCMs.^[^ ^59^ ^]^ As shown in Figure 5c, the thermal storage core material (n‐dodecanol) was self‐assembled into the designed nanoreactor, and acrylate copolymers were further grafted onto the surface of the nanocontainer by in situ polymerization. Microcapsule PCMs with double polymeric shell structures were obtained (Figure 5d). The maximum encapsulation ratio and latent heat reached 90 wt.% and 180 J g^−1^, respectively. In another example, the teams of Zhou reported microcapsule PCMs with a cellulose nanocrystal (CNC)/melamine‐formaldehyde (MF) hybrid shell fabricated via Pickering emulsion polymerization (Figure 5e,f).^[^ ^60^ ^]^ The CNC‐reinforced MF hybrid shell exhibited good mechanical strength and enhanced the PCMs core loading content.

A relatively high PCM core loading weight percentage represents more efficient thermal energy storage performance but poor structural stability, and vice versa.^[^ ^61^ ^]^ Studies related to polynuclear microcapsule PCMs are relatively few, despite their high heat storage efficiency and economics.^[^ ^62^ ^]^ F. Salaün et al. proposed coagulation in situ polymerization method for fabricating multinuclear microparticles.^[^ ^63^ ^]^ In this process, phase change microgel particles were formed by polymerization in a polyvinyl alcohol (PVA)/hexadecane emulsion and further crosslinked together with the addition of isocyanate, then finally encapsulated into a melamine formaldehyde shell. Consequently, the polynuclear microcapsule PCMs exhibited a high latent heat storage density (174 J g^−1^).

In conclusion, the cell‐like structural core‐shell confinement strategy offers an effective way to encapsulate PCMs.^[^ ^64^ ^]^ Moreover, multifunctional PCMs microcapsule platforms have been explored, offering highly promising applications in thermal regulation textiles^[^ ^65^ ^]^ and drug delivery systems.^[^ ^66^ ^]^

#### 3.1.2 Plants‐Like Structure Porous Confinement

**Figure 6:**
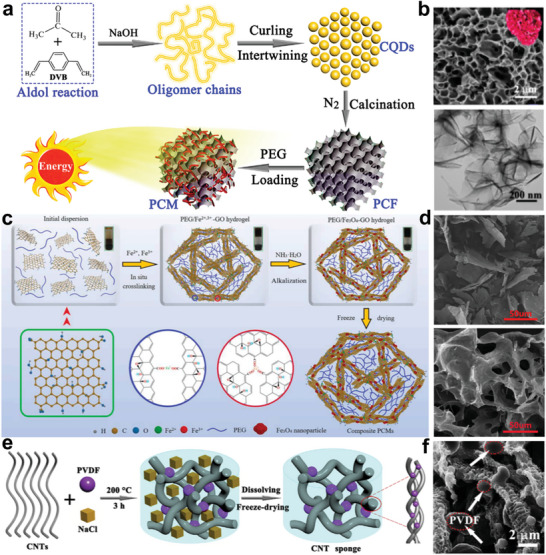
Plants‐like structure porous confinement. a) Schematic illustration of 3D porous carbon composite PCMs. b) Cross‐sectional SEM and TEM images of porous carbon scaffold. Reproduced with permission.^[^ ^68b^ ^]^ Copyright 2018, Elsevier. c,d) Preparation schematic of PEG/Fe_3_O_4_‐GO acoustic‐thermal PCMs and corresponding SEM images. Reproduced with permission.^[^ ^70^ ^]^ Copyright 2021, Elsevier. e,f) Synthetic scheme for PCMs with CNT sponge structure and SEM images. Reproduced with permission.^[^ ^71^ ^]^ Copyright 2020, Elsevier.

Plants in nature are diverse and possess ingenious structures, such as metasequoia with an inner vertical structure^[^ ^67^ ^]^ and sponges with interconnected porous structures,^[^ ^68^ ^]^ which offer inspiration for encapsulating PCMs and enhancing their function.^[^ ^69^ ^]^ Specifically, mesoporous materials have exhibited greater shape stability in PCMs owing to their capillarity and strong interactions. In addition to traditional confinement strategies, natural strategies have made remarkable progress in functional integration by combining mesoporous structures and bionic designs. As shown in **Figure** **6**a, Chen et al. constructed a celosia‐like 3D, highly graphitized thermally conductive carbon network using carbon quantum dots (CQDs).^[^ ^68b^ ^]^ The mean speed and free path of the phonons can be controlled by calcination and cross‐linking reactions. Benefiting from the phonon‐intensive thermal vibration in the sp^2^‐hybridized ordered porous carbon, the thermal conductivity was enhanced by 236%. Additionally, the hyperbranched ordered carbon network provides effective thermally conductive paths and improves the thermal conduction properties of the composites (Figure 6b). Oriented porous carbon provides sufficient pore volume to effectively confine PEG molecules. Eventually, the thermal enthalpy of the composites can reach 160.3 J g^−1^, which is exceedingly close to the theoretical value. Recently, the Tang group developed a PEG/Fe_3_O_4_‐GO phase change aerogel with 3D network structures using freeze‐drying technology and applied it to the acoustic thermal energy conversion field (Figure 6c,d).^[^ ^70^ ^]^ Based on the synergistic effect between the internal friction of the GO network structure and the vibrational heat production of the Fe_3_O_4_ nanoparticles, the PEG/Fe_3_O_4_‐GO composites exhibited efficient acoustic wave absorption and heat energy storage. In a previous study, Chen et al. designed a hierarchically interconnected 3D carbon nanotube (CNT) sponge using an organic‐solvent‐free self‐assembly procedure and impregnated it with PEG to fabricate composite NPCMs (Figure 6e).^[^ ^71^ ^]^ The CNT sponge not only induced capillary action, guaranteeing the stability of the PEG molecules, but also provided numerous heterogeneous nucleation sites for the phase change component (Figure 6f). Consequently, the composites exhibited high thermal energy storage capability and superior thermal stability, assisted by the resulting hierarchical CNT sponge.

In general, the plants‐like structure porous confinement is the most adopted technique in PCMs encapsulation owing to its convenient operation, strong reliability and will work more remarkably in advanced thermal storage systems.^[^ ^40^, ^72^ ^]^

#### 3.1.3 Cobweb‐Like Structure Fibrous Confinement

**Figure 7:**
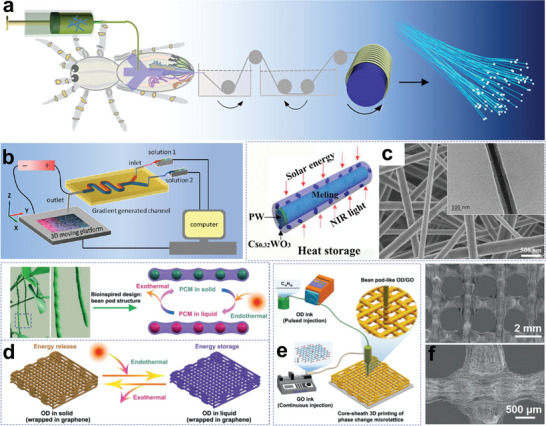
Cobweb‐like structure fibrous confinement. a) Microfluidic spinning technology inspired by spiderwebs. Reproduced with permission.^[^ ^75^ ^]^ Copyright 2021, Elsevier. b) Schematic diagram of the coaxial electrospinning technique. Reproduced with permission.^[^ ^76^ ^]^ Copyright 2016, American Chemical Society. c) Structural schematic of PW@PAN phase change nanofibers and SEM images. Reproduced with permission.^[^ ^77^ ^]^ Copyright 2019, Elsevier. d) Schematic illustration of bean‐pod‐like PCMs and solar‐thermal conversion. e,f) Fabrication procedure of bean‐pod‐like PCMs and SEM images. Reproduced with permission.^[^ ^84^ ^]^ Copyright 2021, Wiley‐VCH.

In nature, spiders can build high‐tensile strength cobwebs using their bodies’ glands to catch prey and escape predators, which has attracted extensive research in the field of artificial fibers.^[^ ^73^ ^]^ Microfluidic technology operates in the same spirit as the essential hydrodynamic principles found in nature and has emerged as a fascinating approach in the continuous production of nanofibers,^[^ ^74^ ^]^ sparking interest in the fabrication of phase change nanofibers (**Figure** **7**a).^[^ ^75^ ^]^ At present, electrospinning, melt spinning, and microfluidic spinning are the most common technologies used for producing phase change nanofibers.

Electrospinning is a technology for drawing ultrafine fibers using electrostatic forces.^[^ ^79^ ^]^ In this way, core‐sheath phase change nanofibers can be achieved by the coaxial electrospinning technique.^[^ ^80^ ^]^ For example, the Xia group first reported the octadecane@TiO_2_‐ polyvinylpyrrolidone (PVP) phase change nanofibers with a core‐sheath structure by the melt coaxial electrospinning method in 2006.^[^ ^81^ ^]^ The basic principle of this technology is shown in Figure 7b, where the core/sheath fluids are fed into the inner/outer capillary by a syringe pump and contacted to form a coaxial Taylor cone.^[^ ^76^ ^]^ Then, they further convert into a coaxial fluid jet in a high‐voltage electrostatic field, forming core‐sheath fibers, which are finally received by the target platform.^[^ ^82^ ^]^ Lu and co‐workers successfully fabricated paraffin wax (PW)@polyacrylonitrile (PAN) thermo‐regulated phase change nanofibers decorated with hexagonal cesium tungsten bronze (Cs_0.32_WO_3_) by coaxial electrospinning technique (Figure 7c).^[^ ^77^ ^]^ In addition to their excellent NIR absorbing ability, the textiles exhibited relatively high latent heat (60.31 J g^−1^) and good shape stability.

The combination of natural structures and bionic manufacturing approaches has remarkably promoted the development of phase change fibers.^[^ ^83^ ^]^ Inspired by the natural bean‐pod structure (Figure 7d), Yang and co‐workers proposed an extrusion‐based core‐sheath 3D‐printing strategy to construct bean‐pod‐like phase change nanofibers and assemble into a phase change micro‐lattice device (Figure 7e,f).^[^ ^84^ ^]^ The closely stacked graphene sheets improved the thermal energy transfer rate. With the combination of light spreading facilitation by the porous lattice structure, the NPCMs exhibited an impressive solar‐thermal energy storage ability, as well as a high heat energy density (190.0 J g^−1^).

**Figure 8:**
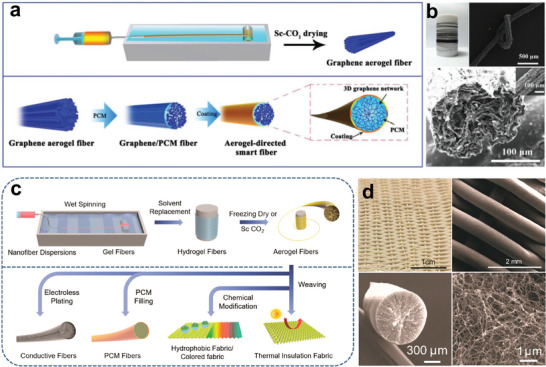
Cobweb‐like structure smart phase change fibers. a) Schematic illustration of the processes of ASF phase change fibers. b) Photograph and SEM images of the phase change fibers. Reproduced with permission.^[^ ^39^ ^]^ Copyright 2018, Wiley‐VCH. c) Schematic showing the preparation of KNF aerogel phase change textiles. d) Photograph and SEM images of the aerogel textile. Reproduced with permission.^[^ ^78^ ^]^ Copyright 2019, American Chemical Society.

The Zhang group conducted numerous studies in the field of bionic aerogel composite fibers. As shown in **Figure** **8**a, the graphene aerogel fibers were first prepared by microfluidic spinning technology and a supercritical fluid drying process.^[^ ^39^ ^]^ The aerogel‐directed smart fibers (ASFs) were fabricated by impregnating PCMs with a subsequent coating of fluorocarbon resin. Significantly, the highly porous 3D graphene network (Figure 8b) endowed the resulting smart fibers with superior mechanical, electrical, and thermal properties. As a result, the final smart fabric exhibited multi‐responsive stimulus properties (electrical/thermal/photonic). As is well known, Kevlar nanofiber (KNF) is an ideal textile fiber with excellent mechanical performance. Given its superiority, bionic KNF‐based phase change fibers were fabricated through wet‐spinning and PEG vacuum impregnation processes (Figure 8c,d).^[^ ^78^ ^]^ Due to the strong capillary force of the porous structure, the aerogel fibers can hold a high percentage of PEG without any leakage, revealing high energy storage properties (162.0 J g^−1^) and form stability. To sum up, cobweb‐like structured fibrous confinement has been the most fascinating technique for PCMs encapsulation in the last decade. With the rapid growth of microfluidic technology, phase‐change fabrics exhibit larger areas as well as controllable sizes and are expected to prevail in the fields of intelligent wear and sensing.^[^ ^7c^ ^]^

**Figure 9:**
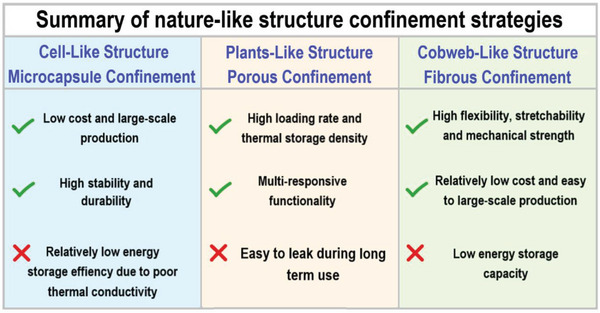
Summary of nature‐like structure encapsulation strategies.

Overall, nature is a rich source of inspiration and has provided a wise route to encapsulation strategies of PCMs. Here, an overview and comparison of nature‐like structure encapsulation strategies are summarized (**Figure** **9**).^[^ ^85^ ^]^ Undoubtedly, natural strategies have effectively promoted the development of PCMs, particularly for multifunction integration. However, there are still deficiencies in natural confinement strategies that need to be further explored.

### 3.2 Nature‐Like Macro Functionality of Thermoresponsive Systems

From ancient times to the present, nature has strongly invoked a desire to explore, thereby promoting the vigorous development of modern bionics.^[^ ^86^ ^]^ As one of the main branches of bionics, the scope of functional bionics is very extensive, covering almost all vital movements of biological organisms. Such as the solar energy utilization systems with the inspiration of green plants' photosynthesis,^[^ ^87^ ^]^ self‐cleaning interface materials imitate superhydrophobic phenomena in nature.^[^ ^88^ ^]^ Specific to the field of PCMs, numerous nature‐like thermoresponsive systems have also been developed.^[^ ^89^ ^]^ In particular, they formed unique living habits especially the self‐protection behaviors of natural organisms during millions of years of evolution. More specifically, Zhang, and co‐workers reported phase transition‐controlled full‐color displays with the inspiration of the chameleon discoloration.^[^ ^90^ ^]^ Additionally, Tang et al. proposed a thermo‐electric system based on PCMs inspired by the electric eel.^[^ ^91^ ^]^ Indeed, bionic functionality has been widely employed in bionic robots, displays, and IR stealth applications. In view of the bionic function design principle, the latest advances in nature‐like thermoresponsive systems are summarized in this section.

#### 3.2.1 Thermal Stimulation Robots of Nature‐Like Functionality

In nature, many plants and animals (e.g., rattlesnakes and *Mimosa pudica*) can sense and respond quickly to their surroundings.^[^ ^92^ ^]^ Inspired by natural actuator behaviors, scientists have made great progress in the development of bionic robots in recent years.^[^ ^93^ ^]^ In this respect, thermal stimuli‐sensitive bionic robots have also been developed by harnessing the reversible volume‐change characteristics of PCMs. Specifically, the supporting materials resemble rigid bones, and the phase change component acts as soft tissue in response to thermal stimulation. Thermal expansion of soft tissues can drive the directional motion of skeletal materials under heat stimulation.^[^ ^94^ ^]^ Generally, a layered structure is effective in designing thermally sensitive robots.^[^ ^95^ ^]^

**Figure 10:**
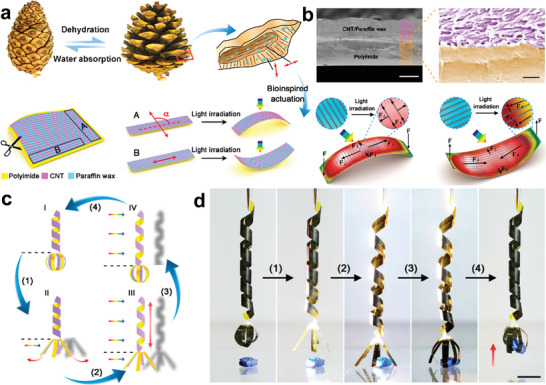
Nature‐like smart robotic arm based on PCMs. a) Schematic showing the fabrication of a pinecone‐inspired PCMs composite film. b) SEM images of the composite film. c,d) Schematic diagram of the actuation process and photographs of the robotic arm responding to light irradiation. Reproduced with permission.^[^ ^32^ ^]^ Copyright 2016, American Chemical Society.

Inspired by the bilayer architecture of pinecones, the Peng group reported an effective strategy for designing thermal stimuli‐sensitive bionic robots, which involved embedding aligned CNTs in PW on a Kapton film (**Figure** **10**a).^[^ ^32^ ^]^ As shown in Figure 10b, the PW was filled into the aligned CNTs, and formed a composite layer compactly contacted the substrate film. When exposed to light irradiation, the PW phase changed and thermally expanded, but was constrained by the aligned CNTs. Then, tensile/contractile stress was produced as an accompanying effect of expansion. As a result, different types of motion arose from the competition between tensile and contractile stress. Based on the mechanism of phototropic bending, a light‐manipulated mechanical arm composed of a telescopic arm and bendable machine claw was assembled using a preprogrammed nanoscale structuring method (Figure 10c). The mechanical arm can manipulate the elongation, release, grasp, and contraction movements in turn, and can grab objects by regulating the illuminated areas (Figure 10d).

**Figure 11:**
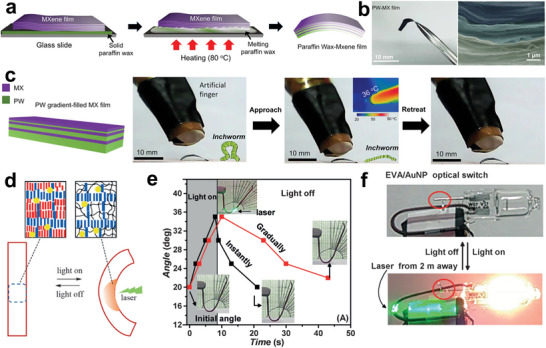
Nature‐like thermal actuators based on PCMs. a) Schematic diagram for preparing the PW‐MX phase change film. b) Photograph and SEM images of PW‐MX phase change film. c) Crawling optical images of the inchworm robot based on PW‐MX phase change film. Reproduced with permission.^[^ ^96^ ^]^ Copyright 2021, American Chemical Society. d) Reversible optical actuation schematic representation of phase change semicrystalline polymers. e,f) Optical actuation photographs of composite exposure to laser and photoelectrical switch application. Reproduced with permission.^[^ ^98^ ^]^ Copyright 2017, Wiley‐VCH.

Additionally, the introduction of new materials has created great opportunities for the development of thermal actuators. Xiao and co‐workers developed an inchworm robot with a structure comprising a gradient PW‐filled Ti_3_C_2_T_x_ (PW‐MX) film (**Figure** **11**a).^[^ ^96^ ^]^ The melt PW spreads out along the vertical direction due to gravity, forming the gradient‐PW‐filled structure (Figure 11b). The robot could bend or extend owing to the stress released during the phase change process inside the PW when subjected to thermal radiation. Benefiting from the multilayer heterostructure, the inchworm robot realized directional locomotion triggered by a human finger (Figure 11c). With the exception of bionic motion, thermal stimuli‐sensitive bionic robots also show potential applications in tunable optical actuators.^[^ ^97^ ^]^ For example, the Zhao group designed photo‐responsive semicrystalline polymers with a network structure and further surface‐functionalized them with Au nanoparticles.^[^ ^98^ ^]^ The bionic robots are composed of skeletal (high *T*_m_) and internal actuation domains (lower *T*_m_). Based on the thermal actuation principle, a contraction/expansion force arises during the phase transition of the actuation domains through the photothermal effect (Figure 11d). As a result, the robots showed temperature‐memory behavior under light irradiation (Figure 11e) and were successfully applied to intelligent optical switch devices (Figure 11f). Although continuous breakthroughs have been achieved in the field of thermal actuators over the last few decades.^[^ ^99^ ^]^ Essentially, the great majority of thermal‐responsive bionic robots remain in the experimental stage.^[^ ^100^ ^]^ The realization of mass production and device highly integration remains a formidable challenge.

#### 3.2.2 Thermoresponsive Display of Nature‐Like Functionality

The patterning and dynamic coloration changes that occur in cephalopod skin (e.g., blue‐ringed octopus, cuttlefish, squid) bring abundant inspiration for the development of intelligent display and interaction technologies.^[^ ^101^ ^]^ This astonishing performance in nature stems from the alternately arranged nanostructures of pigment cells and reflective cells with unique optical functions.^[^ ^102^ ^]^ Coincidentally, the PCMs show variable optical transparency characteristics during melting/crystallization.^[^ ^103^ ^]^ In this regard, numerous thermoresponsive display NPCMs with layered structure have been reported based on the phase transition characteristic.^[^ ^104^ ^]^

**Figure 12:**
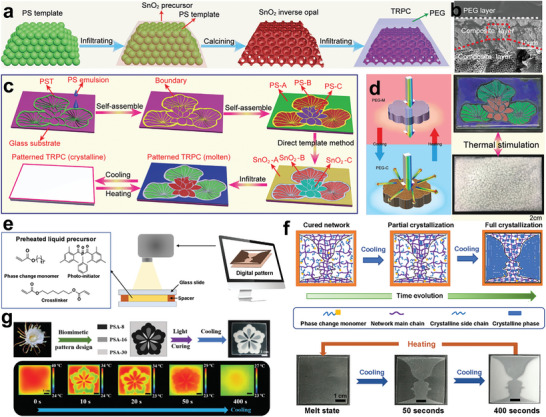
Nature‐like thermoresponsive display devices. a,b) Schematic illustration of the fabrication process for the thermally responsive PEG–SnO_2_ displays and corresponding SEM images showing the layered structure. c) Schematic illustrating the preparation of the patterned thermally responsive displays. d) Operating principle and photograph of the patterned thermally responsive displays. Reproduced with permission.^[^ ^105^ ^]^ Copyright 2020, Wiley‐VCH. e) Digitally programable curing technique for preparing PCMs. f) Schematic diagram showing the fabrication process of digitally programable PCMs along with optical images. g) Optical and IR images showing the evolution of the dual‐mode temporal communication devices. Reproduced with permission.^[^ ^35^ ^]^ Copyright 2021, Wiley‐VCH.

The teams of Tang reported a thermal response display device by infiltrating PEG into an SnO_2_ inverse opal porous nanostructure (**Figure** **12**a).^[^ ^105^ ^]^ As shown in Figure 12b, the device was composed of an upper PEG thermal optical switch and SnO_2_ inverse opal display layers. The optical switch layer controls the optical path by transforming crystalline PEG into an amorphous state. The lotus‐patterned thermally responsive display device was designed via the confinement self‐assembly method, and the lotus pattern enabled presentation/concealment under thermal response (Figure 12c,d). Based on the time‐evolution properties of the variable optical transparency in PCMs, Zhao et al. reported a dual‐mode temporal communication device fabricated using light‐cured programable technology.^[^ ^35^ ^]^ The system is prepared consisting of a phase change monomer (stearyl acrylate), crosslinker (1,6‐hexanediol diacrylate), and photoinitiator by photo‐polymeric process (Figure 12e). In principle, the pattern encryption of the system is realized along with its optical transparency during the phase transition process.^[^ ^106^ ^]^ Meanwhile, information encryption in the time dimension was achieved by encoding temporal information (Figure 12f). As a result, the live evolution of both optical and thermal images for temporal communication was achieved by a dual‐mode temporal system (Figure 12g). Overall, the exploration of thermoresponsive display devices based on NPCMs has facilitated the development of information security.^[^ ^107^ ^]^ Further research is recommended for the integration of devices.

#### 3.2.3 Infrared Radiation Regulatory of Nature‐Like Functionality

Nature's design principles also provide effective solutions for regulating IR radiation in modern society.^[^ ^108^ ^]^ In extremely cold environments or high‐altitude areas, polar bears and yaks use their thick fat covered by hollow and crimped hair to effectively absorb and reflect IR radiation from their bodies, making them invisible, even under an IR camera.^[^ ^109^ ^]^ Inspired by the IR stealth mechanism of polar bears, artificial IR thermal stealth can be achieved by combining adjustable thermal emissivity with thermal management properties.^[^ ^110^ ^]^ Thermal emissivity can be realized by regulating surface micro/nanostructures. On the other hand, the PCMs are an ideal thermal management material owing to their adjustable operating temperature and exceptional thermal storage capacity.^[^ ^111^ ^]^ Therefore, IR stealth technology based on NPCMs has been developed and shows great application potential in high technology and modern military usage.^[^ ^112^ ^]^

**Figure 13:**
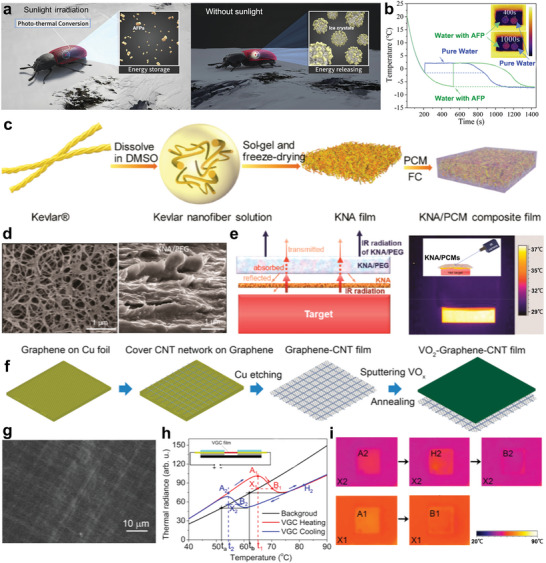
Nature‐like infrared radiation regulatory systems. a) Schematic diagram illustrating the principle behind the antifreezing capability of antifreeze beetles. b) Thermal management capability comparison of pure water and AFP solution. Reproduced with permission.^[^ ^113^ ^]^ Copyright 2022, Elsevier. c,d) Schematic representation of the preparation of KNA/PCMs composite film and SEM image. e) IR stealth mechanism and thermal image of KNA/PCMs composite films. Reproduced with permission.^[^ ^34^ ^]^ Copyright 2019, American Chemical Society. f,g) Schematic of the fabrication process of VGC film and corresponding SEM image. h,i) IR stealth thermal radiance evolutions and images of VGC‐based device. Reproduced with permission.^[^ ^116^ ^]^ Copyright 2015, American Chemical Society.

Antifreeze beetles can survive in extreme environments by relying on antifreeze proteins (AFPs) in their bodies, which act via hydrogen bonding and hydrophobic interactions (**Figure** **13**a).^[^ ^113^ ^]^ Compared with pure water, the AFPs solution exhibits more efficient thermal management and IR stealth capabilities (Figure 13b). Taking inspiration from the discoveries in nature, Zhang et al. fabricated KNA/PCMs IR stealth films with 3D network structures by impregnating PEG into Kevlar nanofiber aerogel (KNA) (Figure 13c).^[^ ^34^ ^]^ As shown in Figure 13d, the Kevlar aerogel with the network structure showed good thermal insulation ability, and the KNA/PCMs composite film achieved a high thermal management capacity (179.1 J g^−1^) and comparable IR emissivity (0.94) to the background after being filled with PEG. Furthermore, IR thermal insulators consisting of a thermal insulation layer (KNA) and IR absorption surface layers (KNA/PCMs) were designed and applied to hot object thermal stealth. With their combination of thermal insulation and IR absorption performance, the hot targets covered with composite thermal insulators became completely invisible under IR detection (Figure 13e). In addition to temperature regulation, controlling and reducing the emissivity of materials is an ideal way to realize IR stealth.^[^ ^114^ ^]^ Vanadium dioxide (VO_2_) is an ideal controlled IR emissivity material and is capable of variable light transmittance in the IR region via its transformation from a low‐temperature monoclinic insulating phase to a rutile metallic phase at high temperatures.^[^ ^115^ ^]^ Recently, Xiao and co‐workers prepared a VO_2_/graphene/CNT (VGC) sandwich‐like thin film capable of electrothermally driven thermal radiation control (Figure 13f,g).^[^ ^116^ ^]^ These composites regulate their transmittance and blend into the surrounding environment via joule heating. Their adaptive thermal camouflage performance was further demonstrated using a VGC‐based thermal camouflage system (Figure 13h,i). With the combination of direct and hysteretic heating strategies, the device realized rapid switchable thermal camouflage. Briefly, the nature‐like thermoresponsive systems have shown a vibrant vitality in intelligent robots, screen displays, and military fields are listed in **Table** **2**.^[^ ^117^ ^]^ Highly intelligent and mass production are still the trends of future development of functional bionics. In essence, the integration of NPCMs with bionic microstructures is an effective route for realizing functional bionics and requires further research.^[^ ^118^ ^]^

**Table 2:** Summary of nature‐like thermoresponsive systems

Classifications	Principles	Promising applications	Tendency
Thermally stimulated robots of nature‐like functionality	Reversible volume changes	Intelligent actuators	Intelligence
			Mass production
Thermoresponsive display of nature‐like functionality	Variable optical transparency	Screen display	Convenience
			Multifunction
IR radiation regulation of nature‐like functionality	Thermal energy absorption	Military defense	Low‐cost
			Mass production

### 3.3 Nature‐Like Structural Design–Function Relationship

**Figure 14:**
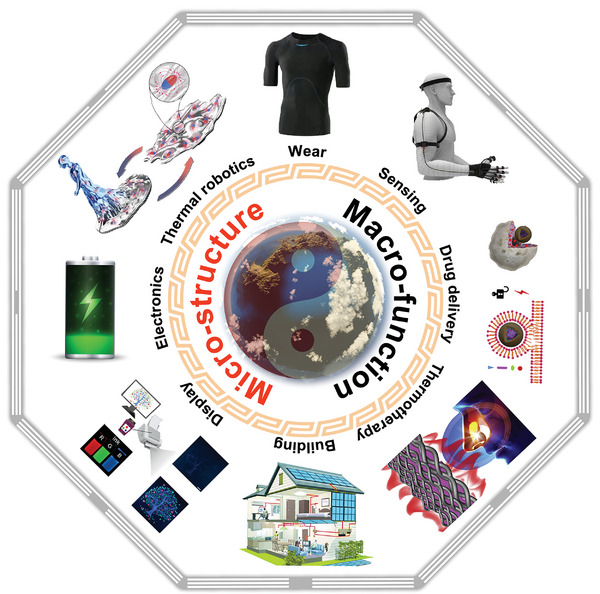
The relationship of bionic structure–function in PCMs with the Tai Chi as a prototype: the common natural origin and complement each other. Emerging applications including: Wear, sensing, drug delivery. Reproduced with permission.^[^ ^136^ ^]^ Copyright 2018, Elsevier. Thermotherapie: Reproduced with permission.^[^ ^71^ ^]^ Copyright 2020, Elsevier. Building, display: Reproduced with permission.^[^ ^90^ ^]^ Copyright 2021, Wiley‐VCH. Electronics, thermal robotics: Reproduced with permission.^[^ ^100b^ ^]^ Copyright 2021, Wiley‐VCH.

As per the ancient Chinese philosophy, the Book of Changes states that Tai Chi, as the “supreme ultimate” of the universe, gave birth to the modes of yin and yang, which form the basis of the entire world.^[^ ^119^ ^]^ Similarly, with inspiration of the nature, NPCMs can be divided into structural and functional materials according to their focus (**Figure** **14**).^[^ ^120^ ^]^ The structural bionics of NPCMs involve imitating the nanostructure of stem cells,^[^ ^43^ ^]^ spider silk,^[^ ^121^ ^]^ or bamboo, and achieving the encapsulation of PCMs based on the nanoconfinement effect.^[^ ^122^ ^]^ Then, numerous bionic nanoconfinement structures, including core‐shell,^[^ ^123^ ^]^ longitudinal,^[^ ^124^ ^]^ and porous structures, have been widely presented.^[^ ^125^ ^]^ Meanwhile, adhesion forces,^[^ ^126^ ^]^ Van der Waals interactions,^[^ ^127^ ^]^ and capillary actions play important roles in this process.^[^ ^128^ ^]^ Nevertheless, the functional bionics of NPCMs focus on bionic functionalization through the integration of functional components.^[^ ^129^ ^]^ With the macro composition of functional structure, multifunctional integration is realized.^[^ ^130^ ^]^ The combination of structural and functional bionics is an effective approach for developing next‐generation NPCMs.^[^ ^131^ ^]^ For instance, inspired by the structure and non‐freezing property of *Lobelia telekii*,^[^ ^132^ ^]^ the He group reported new NPCMs with a solar anti‐icing function via microscopic structural confinement and macro function integration.^[^ ^133^ ^]^ In addition, the appropriate selection of supporting materials can yield twice the result with half the effort. Beneficial from easy grafting and photothermal properties of MXenes,^[^ ^134^ ^]^ The teams Tang reported new NPCMs with a bionic sandwich structure, which achieved superior photothermal storage capability.^[^ ^135^ ^]^

Meanwhile, yin and yang originate from each other.^[^ ^137^ ^]^ This is analogous to the relationship between the structural design and function of NPCMs, which are inextricably linked with and complement each other. Notably, the integration of the structure and function is a frontier in NPCMs research. A successful bionic system requires not only the unity of structure and function but also the coordination between parts and the whole. Therefore, research on the bionic structure–function integration of NPCMs is of great scientific importance and has extensive application prospects in the fields of human motion,^[^ ^138^ ^]^ medicine,^[^ ^139^ ^]^ and intelligent thermal management devices.^[^ ^140^ ^]^

## 4 Emerging Applications

Over the last decade, the research scope of NPCMs has been extensive and actually promoted progress in human society.^[^ ^141^ ^]^ This section presents recent advances in the application of NPCMs.

### 4.1 Wearable Thermal Management Textiles

**Figure 15:**
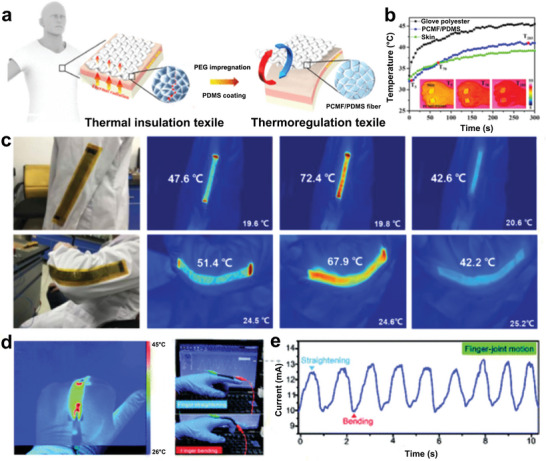
NPCMs for thermal regulation of the human body. a,b) Schematic representation of wearable thermal regulation textiles and thermal management tests. Reproduced with permission.^[^ ^149^ ^]^ Copyright 2020, American Chemical Society. c) Thermal management performance of flexible electrothermal phase change textiles. Reproduced with permission.^[^ ^150^ ^]^ Copyright 2021, Elsevier. d,e) Thermal image and motion‐sensing tests of the phase change textiles. Reproduced with permission.^[^ ^151^ ^]^ Copyright 2019, Royal Society of Chemistry.

NPCMs can also work in vitro for human health via thermotherapy.^[^ ^142^ ^]^ Inspired by the stomatal structures of plants, Chen et al. developed NPCMs with superior thermal harvesting and regulatory properties by infiltrating PEG into a polyethylene sponge.^[^ ^71^ ^]^ Subsequently, a thermotherapy mask based on NPCMs was proposed for thermotherapy, which consisted of an outer air‐purification layer and an inner thermal regulation layer (**Figure** **15**a,b). Not only can the thermotherapy mask capture particulate matter and purify the inhaled air (Figure 15c,d), but it can also exert thermotherapeutic efficacy against allergic rhinitis by heating the airflow into the nasal cavity in the latent heat release process (Figure 15e). Additionally, NPCMs offer unique opportunities for temperature‐controlled drug delivery, benefiting from the adjustable phase change temperature and solid–liquid transition. As classic therapeutic NPCMs, natural fatty acids, fatty alcohols, and their eutectic mixtures have been widely applied in biomedicine owing to their satisfactory biocompatibility and biodegradability.^[^ ^143^ ^]^ Generally, drug molecules and NPCMs are co‐encapsulated in hollow or core‐shell particles, where NPCMs play the part of the gate‐keeper to modulate drug release behaviors.^[^ ^144^ ^]^ Solid‐state NPCMs can restrict the behavior of drug molecules below the phase change temperature. When above the melting point, confined drug molecules can be released via a phase transition process. The Xia group has explored numerous studies on near‐IR‐controlled drug delivery systems based on NPCMs, where hollow polystyrene^[^ ^51^ ^]^ and Au nanocages were introduced as carriers and loaded with NPCMs as well as therapeutic agents,^[^ ^145^ ^]^ thereby realizing precise drug delivery exposure to direct heating or high‐intensity focused ultrasound. In another recent study, Meng, and co‐workers presented an NIR‐responsive and thermo‐regulated drug delivery nanoplatform by encapsulating the drug carriers zeolitic imidazolate framework‐8 (ZIF‐8)/doxorubicin hydrochloride (DOX) composites with the NPCMs and further modifying the composites with polydopamine (PDA) (Figure 15f).^[^ ^136^ ^]^ In this system, the NPCMs not only acted as a drug composite medium but also as a thermal response switch to control the release and diffusion process of the drug. The PDA coating of the composites loads a photothermal transfer agent to trigger the thermal response switch of NPCMs for NIR‐controlled drug release. As a result, the obtained drug delivery system achieved effective chemotherapy for cancer treatment (Figure 15g). In addition, biomimetic phase change nanofibers provide opportunities for the on‐demand delivery and controlled release of drugs, including anticancer drugs, antibiotics, and proteins.^[^ ^146^ ^]^ In principle, the volume of phase change nanofibers changes during phase transition and affects the release behavior of encapsulated drugs. Wang et al. proposed a temperature‐regulated drug‐delivery system based on phase change fibers by loading DOX and lauric acid (LA) into PAN/ZIF‐8 fibers (Figure 15h).^[^ ^147^ ^]^ This system showed satisfactory in vitro controlled drug release under NIR irradiation, thereby achieving mild photothermal therapy and chemotherapy for tumors. Admittedly, PCM‐based controlled release systems have emerged in an endless stream and have shown great potential in cancer treatment.^[^ ^148^ ^]^

**Figure 16:**
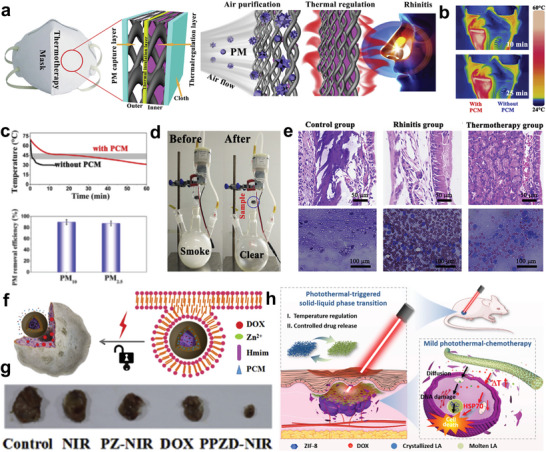
NPCMs for life sciences and medical services. a) Schematic design and working principle of thermotherapy NPCMs mask. b) Thermal infrared images of the mask in actual thermotherapy. c,d) Air‐purification performance and corresponding optical images of the NPCMs mask. e) Representative sections of the nasal tissues of different groups. Reproduced with permission.^[^ ^71^ ^]^ Copyright 2020, Elsevier. f) Schematic diagram of the PDA‐PCM@ZIF‐8/DOX composites. g) Optical images of mice tumor volume in different groups after being subjected to NIR irradiation. Reproduced with permission.^[^ ^136^ ^]^ Copyright 2018, Elsevier. h) Schematic of biomimetic phase change nanofibers for on‐demand drug delivery. Reproduced with permission.^[^ ^147^ ^]^ Copyright 2022, Springer.

Owing to the adjustable thermal regulation and near‐constant temperature during the phase transition process,^[^ ^152^ ^]^ NPCMs have attracted considerable interest in the field of wearable devices. To date, a series of functional thermal regulation textiles have been fabricated by incorporating PCMs with a phase transition temperature of 18–35 °C into fibers,^[^ ^153^ ^]^ fabrics,^[^ ^154^ ^]^ and foams.^[^ ^155^ ^]^ Specifically, Tao et al. filled microstructured fibers with PEG and fabricated wearable thermoregulation textiles after coating them with polydimethylsiloxane (**Figure** **16**a).^[^ ^149^ ^]^ The textiles exhibited exceptional thermal insulation and temperature regulation performance. Ultimately, the textiles achieved better thermoregulation performance than polyester gloves, effectively realizing on‐body intelligent heat management (Figure 16b). Besides, active thermal management methods (e.g., electrothermal energy and solar–thermal conversion) are also effective approaches to personal thermoregulation.^[^ ^156^ ^]^ For instance, Shi, and co‐workers proposed a wearable NPCM device with electro‐thermal energy conversion ability by combining PCMs and graphene films.^[^ ^150^ ^]^ Benefiting from the good conductivity of the graphene film good conductivity and the flexibility of PCMs film, the device achieved an unprecedented electrothermal energy conversion performance (*η* = 94%) and comfortability (Figure 16c). In addition, NPCMs are promising for applications in sports and human health monitoring.^[^ ^157^ ^]^ With the inspiration of the natural fabric structure, the teams of Tang reported a flexible NPCMs film for motion sensing by incorporating carbonized cotton cloth as a conductive supporting scaffold and PW as a thermal regulation component.^[^ ^151^ ^]^ Consequently, the conductive NPCMs demonstrated both thermoregulation and motion‐detection properties (Figure 16e). However, despite the significant progress in intelligent thermal regulation textiles, additional attempts should be made to improve comfort and mass production.^[^ ^158^ ^]^

### 4.2 Life Sciences and Medical Service

**Figure 17:**
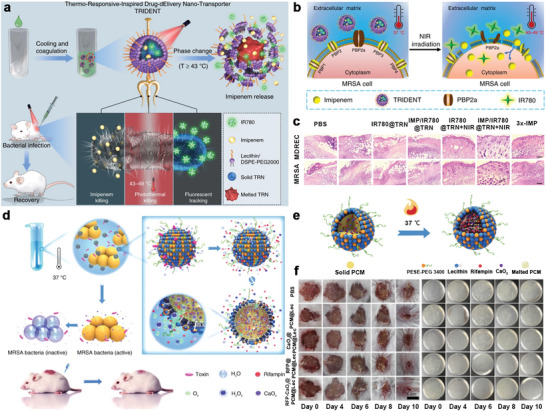
NPCMs for therapy of bacterial infections. a) Schematics illustrating efficient chemo‐photothermal therapy. b) Working principle of bacterial infection therapy. c) Histological photomicrographs of mice skin tissue sections after antibacterial experiments. Reproduced with permission.^[^ ^159^ ^]^ Copyright 2019, Springer Nature. d) Schematic illustration of liposome‐based nanoreactors for bacterial infection therapy. e) Scheme for the antibiotic release process. f) Photographs of wounds and bacteria inhibitory tests. Reproduced with permission.^[^ ^160^ ^]^ Copyright 2019, Springer Nature.

PCM‐based targeted drug delivery systems have also pushed for advancements in antimicrobial therapy, as they allow bacterial infections to be treated with minimal antibiotic doses. Recently, Luo and co‐workers reported a thermo‐responsive‐inspired drug‐delivery nanotransporter (TRIDENT) for the efficient therapy of bacterial infections (**Figure** **17**a).^[^ ^159^ ^]^ The TRIDENT system was composed of hydrophobic PCMs (SA, LA), antibiotic imipenem (IMP), and fluorescent dye (IR780), and then encapsulated with lecithin and DSPE‐PEG 2000. The phase transition of PCMs not only results in the antibiotic release under NIR irradiation but also facilitates permeation into target bacteria (Figure 17b). Thus, the diseased tissues gradually recovered owing to the synergistic effects of fluorescence monitoring and chemo‐photothermal combined targeted therapy (Figure 17c). By comparison, active PCM‐based targeted drug delivery systems possess more convenient and extensive application prospects, with a phase change temperature close to that of the human body. Han et al. proposed an endogenous stimulus‐powered targeted drug delivery strategy for the treatment of bacterial infections (Figure 17d).^[^ ^160^ ^]^ Antibiotics together with CaO_2_ were encapsulated in eutectic PCMs with a phase change temperature of 37 °C. And then RFPCaO_2_@PCM@Lec nanoreactors were obtained after coating with liposomes. During treatment, the nanoreactors anchor onto the surface of the target bacteria at 37 °C, whereupon antibiotics are released during the phase transition process and entry of H_2_O molecules (Figure 17e). As anticipated, the nanoreactors exhibited high in vivo antibacterial activity and could effectively treat bacterial infections (Figure 17f). Virtually, medical research related to NIPCMs is still mainly in the primary stages, and it is anticipated that smarter and more intelligent thermotherapy or drug delivery systems will be designed in the future.^[^ ^161^ ^]^

### 4.3 Intelligent Thermal Management Devices

**Figure 18:**
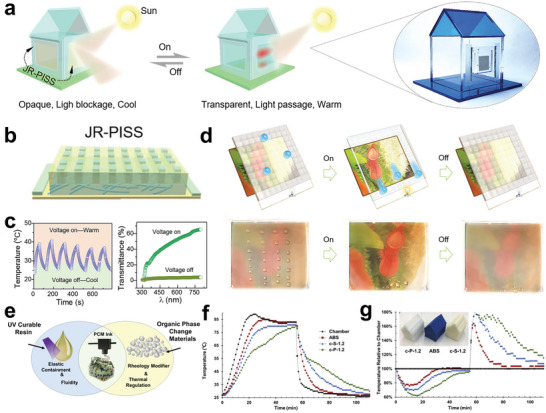
NPCMs for intelligent thermal management buildings. a) Conceptual diagram of a thermal management building with a bioinspired intelligent window. b) Schematic illustration of the joule‐heat‐responsive smart window structure. c) Surface temperature and optical transparency changes of the smart window. d) Optical and wetting performance of the actual tuning surface of the smart window. Reproduced with permission.^[^ ^166^ ^]^ Copyright 2021, Wiley‐VCH. e) Schematic diagram of 3D printing technology for thermal regulation buildings. f,g) The inside temperature variation of the buildings during the heating and cooling process. Reproduced with permission.^[^ ^168^ ^]^ Copyright 2021, Elsevier.

According to statistics from the International Energy Agency,^[^ ^162^ ^]^ the energy consumption of buildings accounts for over 30% of the total energy consumption in the world and is growing along with climate change as well as population growth.^[^ ^163^ ^]^ Hence, intelligent thermal management buildings have drawn intense attention in both scientific and industrial communities.^[^ ^164^ ^]^ Moreover, PCMs are ideal thermoregulation materials that can be incorporated into windows, floors, and other building structures.^[^ ^165^ ^]^ Inspired by the reversibly switchable wettability of Nepenthes, Chen, and co‐workers reported a joule‐heat‐responsive smart window by infusing paraffin into a superhydrophobic micropillar‐arrayed membrane embedded in a silver nanowire thin‐film heater (**Figure** **18**a,b).^[^ ^166^ ^]^ According to the phase transition of paraffin due to the joule‐heating effect, the surface wettability of the device changed from superhydrophobic to superhydrophilic at an applied voltage of 6 V (Figure 18d). Simultaneously, the optical visibility reversibly switched between opaque and transparent states (Figure 18c). Consequently, the concept of smart windows was verified and was successful in building thermal management. Additionally, the 3D printing strategy, as a novel approach, can also promote the development of thermal management devices with complex structures.^[^ ^167^ ^]^ Emily B. Pentzer et al. produced buildings with thermal energy regulation via 3D direct ink printing technology (Figure 18e).^[^ ^168^ ^]^ The printed houses exhibited superior thermal buffer performance with tiny temperature fluctuations during the heating/cooling process compared with the house without PCMs (Figure 18f,g). This 3D‐printing strategy can not only decrease manufacturing costs but also facilitate the production of components with complex geometric structures, providing a facile pathway for intelligent thermal management devices.^[^ ^169^ ^]^

**Figure 19:**
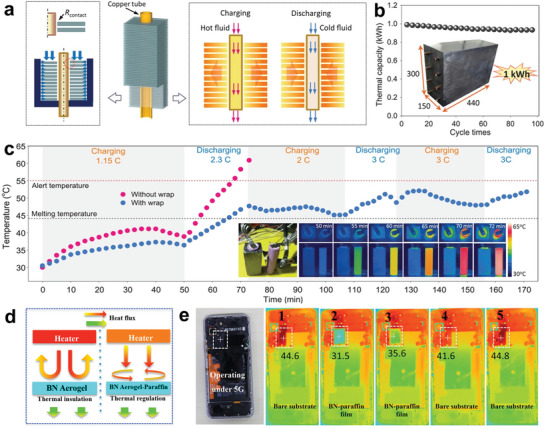
NPCMs for thermal regulation of electronic devices. a) Schematic illustration of the battery thermal energy harvesting device. b) Thermal capacity stability of the device. c) Temperature evolution and IR images of the device inside the battery during the discharging process. Reproduced with permission.^[^ ^174^ ^]^ Copyright 2019, Wiley‐VCH. d) Schematic illustrating the principle of thermal management in portable electronics. e) Thermal infrared images of the portable electronics’ thermal regulation. Reproduced with permission.^[^ ^176^ ^]^ Copyright 2020, American Chemical Society.

Excessive heat accumulation is one of the most common blasting fuses of electronic device circuit failure.^[^ ^170^ ^]^ Therefore, thermal management technology in the electronic device area is indispensable with its miniaturization and integration development.^[^ ^171^ ^]^ NPCMs are promising candidates for electronic thermal management owing to their isothermal phase transition process, which provides an efficient and safe operating environment.^[^ ^172^ ^]^ Typically, the thermal regulation of external packing components composed of PCMs is convenient and effective.^[^ ^173^ ^]^ The teams of Li introduced a compression‐induced method to develop NPCMs with a large‐size aligned graphite sheet structure.^[^ ^174^ ^]^ The composites exhibited a high thermal conductivity (4.4–35.0 W m^−1^ K^−1^), as a result of their highly bionic oriented structure. Subsequently, a high‐power‐density energy harvesting device was designed for power battery thermal management (**Figure** **19**a). The temperature of the wrapped battery monomer was uniformly distributed and could remain below 55 °C at high charging/discharging rates, effectively prolonging the working time of the battery (Figure 19b,c). Besides, the NPCMs film could be effectively used to thermally regulate portable electronics.^[^ ^175^ ^]^ Zhang et al. reported a smart thermal regulator consisting of BN aerogel films and paraffin.^[^ ^176^ ^]^ With a combination of the BN aerogel's thermal insulation effect and the thermal buffering effect of the paraffin phase transition (Figure 19d), the BN–paraffin composite film can effectively dissipate excessive heat generated by electronic devices and promise a beneficial operation environment (Figure 19e). Ultimately, despite numerous high‐performance thermal regulation components based on NPCMs have been applied in electronics or battery thermal management, advanced energy storage systems coupling with NPCMs remain further explored.^[^ ^177^ ^]^

### 4.4 Emerging Energy Conversion Devices

**Figure 20:**
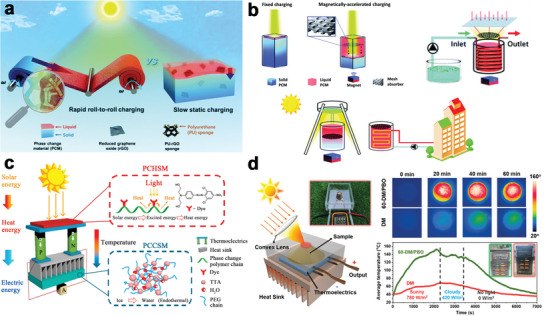
NPCMs for emerging energy conversion devices. a) Roll‐to‐roll solar‐thermal energy harvesting systems. Reproduced with permission.^[^ ^178^ ^]^ Copyright 2020, Royal Society of Chemistry. b) Schematic of magnetically driven solar‐thermal energy conversion devices. Reproduced with permission.^[^ ^180^ ^]^ Copyright 2019, Royal Society of Chemistry. c) Sunlight‐driven thermal energy conversion and generation devices. Reproduced with permission.^[^ ^91^ ^]^ Copyright 2018, Elsevier. d) Schematic of the STEG system for solar energy harvesting. Reproduced with permission.^[^ ^181^ ^]^ Copyright 2022, Wiley‐VCH.

The exploration of energy conversion devices is always eye‐catching with the development of NPCMs. Especially since numerous energy conversion routes have been proposed, such as electro‐to‐thermal,^[^ ^151^ ^]^ photo‐to‐thermal,^[^ ^71^ ^]^ magnetic‐to‐thermal,^[^ ^179^ ^]^ and acoustic–thermal routes.^[^ ^70^ ^]^ Generally, research on solar‐driven systems has been the most extensive due to their wide availability and ease of promotion. However, the utilization of solar energy is limited by discontinuities and instability. These shortcomings can be significantly overcome by combining them with PCMs. Consequently, numerous solar‐driven NPCMs‐based energy conversion devices have been reported in recent years. The Deng group developed numerous efficient solar thermal conversion devices. Inspired by the dynamic thermoregulation behavior of butterfly wings, flexible NPCMs with light absorption capability were designed as a mechanical roll‐to‐roll solar thermal harvesting device that can continuously charge and discharge (**Figure** **20**a).^[^ ^179^ ^]^ As a result, the system achieved efficient and uniform solar‐thermal energy conversion owing to the reduced heat accumulation and increased irradiation area. In another case, a magnetically responsive photothermal mesh was introduced into the PCMs, and a magnetically driven direct solar‐thermal conversion device was developed (Figure 20b).^[^ ^180^ ^]^ Specifically, the mesh could be moved dynamically to accelerate the charging process under a magnetic field, enabling continuous photothermal conversion. Besides the direct solar‐to‐thermal conversion, the solar‐to‐heat‐to‐electric energy conversion route is promising through the integration of PCMs and thermoelectric modules. For instance, solar‐driven phase‐change heat storage materials and phase‐change cool storage materials were applied to the hot/cold sides of thermoelectric systems to achieve solar‐thermal‐electric conversion (Figure 20c).^[^ ^91^ ^]^ Nonetheless, the output electricity of the devices remained at a low level. Then the teams of Fu proposed PCMs with a multi‐directionally arranged structure and achieved an efficient solar‐thermal‐electric conversion device with the utilization of a solar concentrating strategy (Figure 20d).^[^ ^181^ ^]^ And power density of the device reached 198.70 W m^−2^, which is comparable to that of commercial photovoltaic devices. Overall, the practical use of these systems is still in its infancy. It is expected that the NPCMs‐based energy conversion devices are of promising potential in the low‐carbon energy field and encourage further exploration.

## 5 Conclusions and Future Prospects

**Figure 21:**
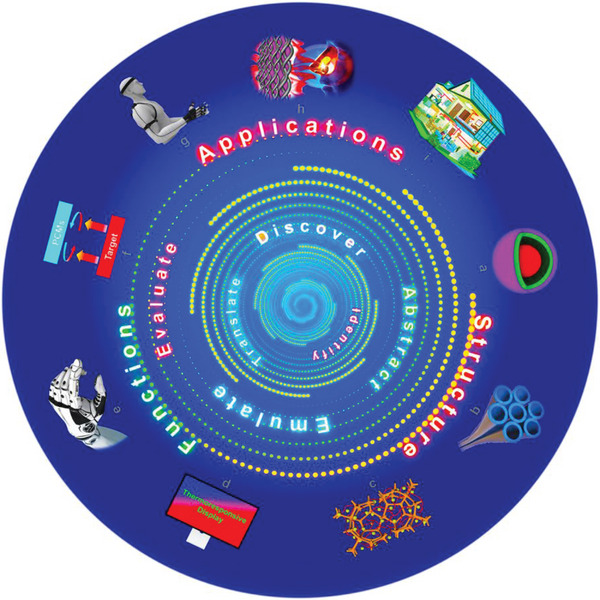
The biomimicry design spiral of the NPCMs. The NPCMs are advancing around the biomimicry design spiral. Structures: a) Cell‐like structure. b) Cobweb‐like structure. Reproduced with permission.^[^ ^78^ ^]^ Copyright 2019, American Chemical Society. c) Plants‐like structure. Functions: d) Display. e) Smart robotics. f) Infrared stealth. Applications: g) Wear and sensing. h) Life sciences and medical service. Reproduced with permission.^[^ ^71^ ^]^ Copyright 2020, Elsevier. i) Intelligent buildings.

The development of bionic materials is always eye‐catching and has penetrated various fields since the 1990s.^[^ ^182^ ^]^ Actually, the development of NPCMs is a step‐by‐step process for turning natural strategies into innovative solutions exactly as the biomimicry design spiral proposed by Carl Hastrich, including identifying, translating, and discovering until final application (**Figure** **21**).^[^ ^183^ ^]^ Accordingly, this review summarizes the pioneering and representative progress in the development of NPCMs for advanced thermal energy management and regulation, from structural design to novel manufacturing and smart TES systems. Moreover, the relationship between the structure and function of NPCMs is discussed to provide basic guidelines for their structural design. According to this, the present limitations and future prospects of NPCMs are investigated in this section.

### 5.1 Inferences

With developments in materials science, chemistry, molecular biology as well as nanotechnology. Nature will take more inspiration for the exploration of next‐generation NPCMs: i) Novel structural designs and encapsulation methods of NPCMs. The thermal properties of state‐of‐the‐art NPCMs require further improvement and it has countless ties with their structural design. Hence, the exploration of new structures could be carried out by mimicking other well‐organized natural products that emphasize the optimal balance between thermal absorption, heat storage, and heat conduction. Significantly, imitation objects are probably not single and can be considered the integration of various local bionic design optimizations aimed at using the smallest quantity of the most highly efficient supporting materials to perform encapsulation and heat transfer functions. ii) Development of multifunctional smart NPCMs. The concept of smart NPCMs refers to advanced PCMs with intelligent attributes or even vital organism characteristics such as sensory feedback, information identification, and self‐regulation abilities. And it has been confirmed as an effective way to obtain smart NPCMs by introducing information science into the designation and manufacture of PCMs. Moreover, nature as a rich source would take rich inspiration for the development of smart NPCMs with bionic technological advancements, such as self‐cleaning NPCMs inspired by the surface microstructure of lotus leaves and the solar‐thermal conversion TES devices with the inspiration of plant's photosynthesis. Undoubtedly, learning from nature offers a significant approach to functionalization, particularly for the intelligentization of NPCMs. iii) Integration of advanced TES systems. Recently, research on TES devices has newly‐emerged. For instance, the Deng group proposed an efficient solar thermal energy storage system with magnetically‐movable optical charging ability and achieved fast solar energy harvesting performance.^[^ ^180^ ^]^ However, the exploration of highly integrated smart TES devices is a general trend and still requires tremendous efforts before practical applications. Natural systems can offer wise guidance for their development. Inspired by the perceived responsiveness of biological organisms, advanced TES systems capable of reacting after sensing a specific signal can be developed, thus achieving rational thermal energy transformation and distribution. Predictably, advanced TES systems with self‐management, adaptive, and protective capabilities could spring up like mushrooms in the near future.

### 5.2 Limitations

Bionic design concepts of NPCMs emerge in an endless stream, putting forward numerous requirements for production technology. Hence, some new solutions have been introduced for the preparation of NPCMs,^[^ ^184^ ^]^ such as electrospinning or 3D‐printing technologies. Despite all this, there are some obstacles to the large‐scale processing of NPCMs, especially for establishing a manufacturing technology with low cost and high durability. Currently, solution mixing and melt compounding are effective processing methodologies for the mass production of phase change fibers, films, and blocks, offering guidance for their commercialization.^[^ ^185^ ^]^ However, these methods have difficulty in achieving accurate microstructure construction. Even for some simple nature‐like microstructures, let alone more complicated structures. Thus, the large‐scale production of materials with precise structures is crucial for high‐performance NPCMs.^[^ ^186^ ^]^ Most NPCMs are still in the experimental stage, and the development of advanced processing technologies can bridge the gap between laboratories and factories. Different from the structural and functional bionics just imitating nature, practical applications require multidisciplinary collaboration as well as dedicated efforts over a long period of time. In addition to exhibiting good application potential in the thermal management of electronics, human bodies, and buildings, NPCMs are ideal candidates for thermal regulation in cutting‐edge fields that require integration with advanced systems or devices for practical applications. Nevertheless, the rational design of integrated systems is a bottleneck, and significant efforts are required. Moreover, special NPCMs, such as those with super flexibility or strong mechanical properties, do not achieve satisfactory performance, restricting their realistic application in the fields of aerospace and military.^[^ ^187^ ^]^ Therefore, the integration of advanced systems and the exploration of new NPCMs are of nearly equal importance for practical applications.

Looking into the future, learning, imitating, and eventually surpassing nature would be the most effective ways to promote NPCMs development.^[^ ^188^ ^]^ In addition to focusing on bionic structural and functional designs, the biological manufacturing process in nature provides an attractive approach from a long‐term perspective. The research of effective bionic processing technology has bright prospects in terms of cost and scale. Despite the coexistence of opportunities and challenges, it is certain that the current progress in NPCMs is only the tip of the iceberg. Significantly, that is a step‐by‐step process for turning natural strategies into sustainable design solutions for NPCMs. Therefore, the exploration and application of novel NPCMs require close interdisciplinary cooperation among chemists, materials scientists, mechanical engineers, and other experts.

## Conflict of Interest

The authors declare no conflict of interest.
